# Inhibition of Striatal Soluble Guanylyl Cyclase-cGMP Signaling Reverses Basal Ganglia Dysfunction and Akinesia in Experimental Parkinsonism

**DOI:** 10.1371/journal.pone.0027187

**Published:** 2011-11-02

**Authors:** Kuei Y. Tseng, Adriana Caballero, Alexander Dec, Daryn K. Cass, Natalie Simak, Elizabeth Sunu, Michael J. Park, Shannon R. Blume, Stephen Sammut, Diana J. Park, Anthony R. West

**Affiliations:** 1 Department of Cellular and Molecular Pharmacology, Rosalind Franklin University/The Chicago Medical School, North Chicago, Illinois, United States of America; 2 Department of Neuroscience, Rosalind Franklin University/The Chicago Medical School, North Chicago, Illinois, United States of America; National Institutes of Health, United States of America

## Abstract

**Objective:**

There is clearly a necessity to identify novel non-dopaminergic mechanisms as new therapeutic targets for Parkinson's disease (PD). Among these, the soluble guanylyl cyclase (sGC)-cGMP signaling cascade is emerging as a promising candidate for second messenger-based therapies for the amelioration of PD symptoms. In the present study, we examined the utility of the selective sGC inhibitor 1H-[1], [2], [4] oxadiazolo-[4,3-a]quinoxalin-1-one (ODQ) for reversing basal ganglia dysfunction and akinesia in animal models of PD.

**Methods:**

The utility of the selective sGC inhibitor ODQ for reversing biochemical, electrophysiological, histochemical, and behavioral correlates of experimental PD was performed in 6-OHDA-lesioned rats and mice chronically treated with MPTP.

**Results:**

We found that one systemic administration of ODQ is sufficient to reverse the characteristic elevations in striatal cGMP levels, striatal output neuron activity, and metabolic activity in the subthalamic nucleus observed in 6-OHDA-lesioned rats. The latter outcome was reproduced after intrastriatal infusion of ODQ. Systemic administration of ODQ was also effective in improving deficits in forelimb akinesia induced by 6-OHDA and MPTP.

**Interpretation:**

Pharmacological inhibition of the sGC-cGMP signaling pathway is a promising non-dopaminergic treatment strategy for restoring basal ganglia dysfunction and attenuating motor symptoms associated with PD.

## Introduction

Currently available pharmacotherapies for PD such as levodopa (L-DOPA) subdue motor symptoms via activation of striatal dopamine (DA) transmission. However, repeated L-DOPA treatment can cause severe side effects (e.g. dyskinesias), most likely as a result of abnormal changes in DA receptor expression and function [1]. Thus, there is clearly a necessity to identify novel non-dopaminergic mechanisms as new therapeutic targets for PD. Among these, the soluble guanylyl cyclase (sGC)-cGMP signaling pathway is emerging as a promising target candidate for treatment strategies aimed at restoring striatal dysfunction induced by DA cell loss [2].

It is now well accepted that sGC is the primary receptor for the gaseous neuromodulator nitric oxide [3]. Interestingly, sGC expression and activity are reportedly higher in the striatum than in any other brain region [4], [5]. At the cellular level, the sGC-cGMP-PKG system is predominantly localized to striatal medium-sized spiny projection neurons (MSNs) of both the direct and indirect output pathways [5], [6], [7]. Until recently, the physiological function of sGC-cGMP signaling in the striatum was unclear. However, numerous recent studies now indicate that sGC-cGMP signaling is likely to function as an important cellular intermediary for regulating interactions between DA and glutamate neurotransmission in the normal and parkinsonian striatum [8], [9], [10], [11], [12], [13]. In fact, findings from studies of animal models of PD indicate that following DA depletion, alterations in striatal cGMP homeostasis are likely to contribute to pathophysiological changes in basal ganglia circuits observed in PD [2]. Specifically, an upregulation of striatal sGC expression and activity (i.e., cGMP synthesis) has been observed in MPTP-treated mice [14], [15]. Interestingly, transient elevations in intracellular cGMP markedly increase striatal neuronal excitability and facilitate excitatory corticostriatal synaptic transmission [12], [16], [17], an effect resembling that observed following chronic DA cell loss [18]. Therefore, we hypothesized that downregulation of the sGC-cGMP signaling pathway should restore pathological changes observed in the basal ganglia after chronic DA depletion, and consequently, reverse motor impairments associated with PD. Towards this goal, we examined the utility of the selective sGC inhibitor 1H-[1], [2], [4] oxadiazolo-[4,3-a]quinoxalin-1-one (ODQ) [19], [20] in reversing biochemical, electrophysiological, histochemical, and behavioral correlates of experimental PD observed in 6-OHDA-lesioned rats and mice chronically treated with MPTP.

## Results

**Figure 1:**
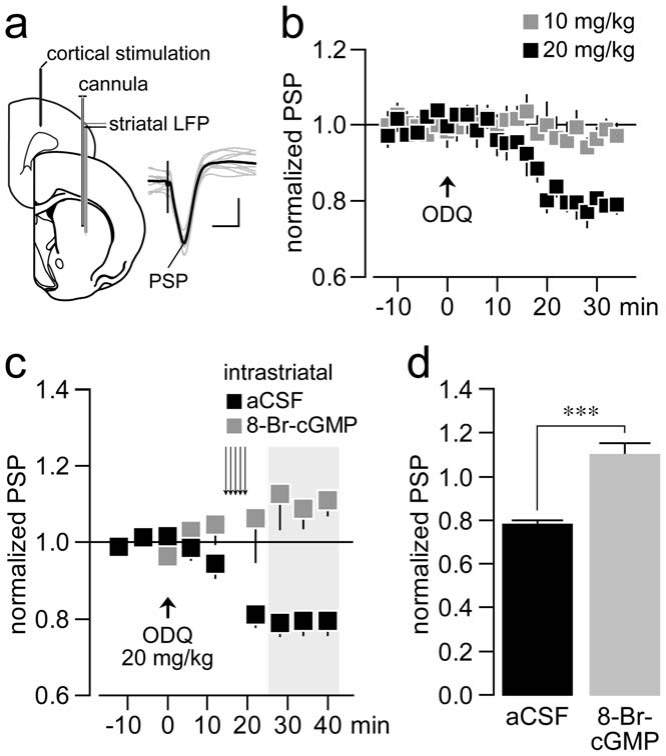
Impact of tonic cGMP signaling on corticostriatal synaptic transmission *in vivo*. (**a**) Recording arrangement employed to study the pharmacological effects of the sGC inhibitor ODQ on corticostriatal transmission *in vivo* (see Methods section for details). Cortically-evoked postsynaptic potentials (PSPs) were recorded by means of local field potential (LFP) recordings. Inset shows examples of traces of corticostriatal PSPs (calibration bars: 30 ms, 1 µV). (**b**) Time course of corticostriatal PSPs recorded before and following systemic administration of 10 mg/kg and 20 mg/kg ODQ (i.p., n = 5 rats per dose). A marked attenuation of the corticostriatal response was observed following 20 mg/kg ODQ, an effect that becomes apparent after 20 min of drug administration. (**c**) Time course of corticostriatal PSPs recorded before and following 20 mg/kg ODQ + intrastriatal administration (0.1 µl/min×10 min) of the cGMP analog 8-Br-cGMP (20 mM; n = 5 rats) or vehicle (aCSF; n = 5 rats). Note that the characteristic attenuation of corticostriatal PSPs observed after 20 min of 20 mg/kg ODQ administration was lacking following intrastriatal infusion of 8-Br-cGMP. (**d**) Bar graph depicting the averaged changes in PSP responses obtained from the last 3 data points shown in **c** (marked in gray). Intrastriatal infusion of 8-Br-cGMP completely blocked the effects of ODQ (***P<0.0005, unpaired t-Test).

We first examined the impact of tonic cGMP signaling on corticostriatal synaptic transmission *in vivo* in naïve rats by measuring changes in cortically-evoked striatal local field potential (LFP) following systemic administration of the selective sGC inhibitor ODQ (Fig 1a;b). Consistent with previous studies [12], systemic administration of ODQ reduced the strength of corticostriatal transmission in a dose dependent manner (Fig 1b). Specifically, a clear attenuation of the corticostriatal postsynaptic potential (PSP) was observed 20 min after administration of 20mg/kg, but not 10 mg/kg dose of ODQ (Fig 1b–c). This inhibition of the cortically-evoked PSP was completely restored by local (intrastriatal) infusion of the cGMP analog 8-bromoguanosine 3′:5′ -cyclic monophosphate sodium salt (8-Br-cGMP) (Fig 1c–d). Thus, as shown in previous studies [17], the ODQ-induced attenuation of corticostriatal synaptic transmission is mediated by local inhibition of striatal sGC and cGMP signaling. The remaining studies described below focused on assessing the utility of ODQ for reversing abnormal striatal neuronal firing activity, basal ganglia dysfunction, and akinesia observed in animal models of PD.

**Figure 2:**
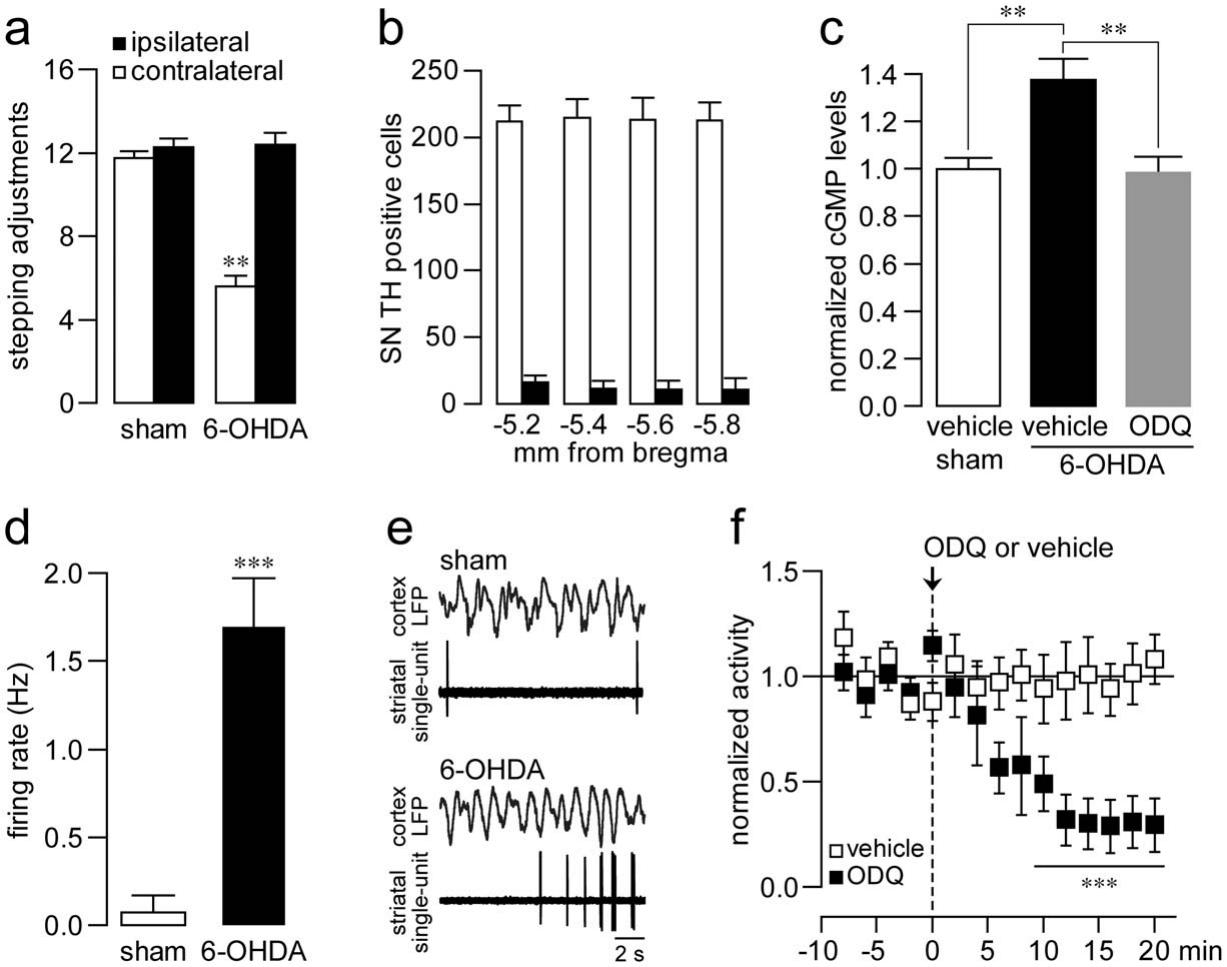
Systemic administration of the sGC ODQ reverses the increased striatal cGMP levels and the elevations in striatal activity observed in 6-OHDA-lesioned rats. (**a**) Behavioral assessment of 6-OHDA-induced unilateral nigrostriatal DA cell lesions. Compared to sham-operated controls (n = 11), all 6-OHDA-lesioned rats included in this study (n = 19) exhibited significant impairments in contralateral forelimb adjustment steps (***P<0.0005, unpaired t-Test), (**b**) Cell counting of TH positive neurons in the SN at four anatomical levels indicated that the degree of DA cell loss observed in all 6-OHDA-lesioned rats included in this study was >90%. (**c**) Striatal tissue cGMP levels were assessed in sham and 6-OHDA-lesioned rats pretreated with vehicle (n = 5–6 per group) or the sGC inhibitor ODQ (20 mg/kg, i.p.). Chronic DA depletion increased striatal cGMP levels (**P<0.01 as indicated, Tukey post-hoc test after significant one-way ANOVA) in a manner that was reversed following a single ODQ treatment (1 hour post injection). (**d**) Bar graph summarizing the increased firing activity of striatal MSNs recorded from 6-OHDA-lesioned rats (n = 25 cells/19 rats) relative to sham-operated controls (n = 13 cells/11 rats) (***P<0.001, unpaired t-Test). (**e**) Electrophysiological traces of simultaneously recorded striatal single-unit activity and cortical LFPs in a sham and a 6-OHDA-lesioned rat. (**f**) Time course showing the effects of one systemic administration of vehicle (n = 5 cells from 5 rats) or ODQ (20 mg/kg, i.p., n = 7 cells from 7 rats) on striatal single-unit activity recorded from 6-OHDA-lesioned rats. ODQ (but not vehicle) markedly reduced elevations in firing rate observed in cells recorded from 6-OHDA-lesioned rats (***P<0.0005, compared to pre-ODQ activity or to measures taken at identical time points as indicated, Tukey post-hoc test after significant ANOVA).

All 6-OHDA-lesioned rats included in the present study exhibited a marked reduction in adjusting steps executed by the contralateral forelimb (Fig 2a), and >90% depletion of DA cells in the substantia nigra (SN), as revealed by TH immunostaining (Fig 2b). We also determined the impact of 6-OHDA-induced DA depletion on striatal sGC-signaling by assessing changes in tissue cGMP levels. We found that chronic DA depletion significantly increased striatal tissue levels of cGMP by ∼35%, an effect that was reversed by one systemic administration of ODQ (20 mg/Kg, i.p., Fig 2c). We next examined whether systemic administration of ODQ also reverses the abnormal striatal electrophysiological activity observed after chronic DA depletions. Consistent with previous studies [18], [21], striatal single-units recorded in neurons from 6-OHDA-lesioned rats exhibited increased spontaneous firing (1.69±0.28 Hz) compared to neurons recorded in sham-operated controls (0.07±0.01 Hz) (Fig 2d–e). We found that one systemic administration of the selective sGC inhibitor ODQ (20 mg/kg, i.p.) robustly decreased the spontaneous firing observed in the DA-depleted striatum (Fig 1f). Specifically, a significant decrease in striatal firing rate from 1.72±0.49 to 0.67±0.43 Hz was observed 10 min after ODQ administration. Vehicle injection did not affect similar measures taken in 6-OHDA-lesioned rats (Fig 2f). Furthermore, ODQ administration did not affect the firing rate of striatal neurons recorded in sham-operated controls (*data not shown*). Together with the biochemical data, these results indicate that an upregulation of sGC signaling may contribute to the abnormal elevation of neuronal excitability observed in the DA-depleted striatum.

**Figure 3:**
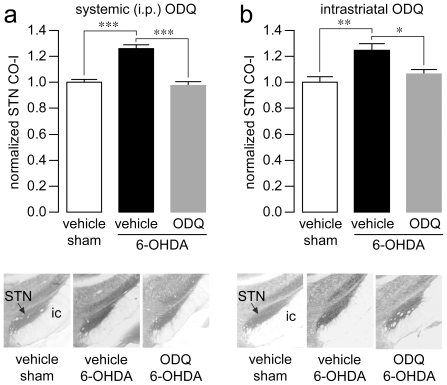
Role of the indirect striatopallidal pathway in mediating the ODQ-dependent reversal of STN hyperactivity. (**a**) Bar graph showing levels of CO-I staining in the STN of sham-operated and 6-OHDA lesioned rats following systemic administration of vehicle or ODQ (20 mg/kg, i.p.). ODQ significantly reduced the increase in CO-I levels observed in the STN of 6-OHDA-lesioned rats (n = 10–11 rats/group; ***P<0.0005 as indicated, Tukey post-hoc test after significant one-way ANOVA). *Inset:* Examples of coronal sections of STN derived from CO-I staining showing the reversal effect of ODQ. (**b**) Summary of the effect of single intrastriatal infusion (0.1 µl/min for 10 min) of vehicle (0.5% DMSO in aCSF) or ODQ (50 µM) on STN CO-I staining. Intrastriatal ODQ also normalized the increased STN CO-I observed in 6-OHDA-lesioned rats (n = 5–7 rats/group; *P<0.05, **P<0.005 as indicated, Tukey post-hoc test after significant one-way ANOVA). *Inset:* Sections of CO-I staining showing the reversal effect of intrastriatal ODQ on STN activity.

STN hyperactivity is another hallmark of parkinsonism as abnormal electrophysiological and metabolic changes have been consistently reported across different animal models of PD and in studies of PD patients [22]. L-DOPA treatment also normalizes STN hyperactivity [23] and mimics the therapeutic effects of STN inactivation/lesion on motor deficits observed in both PD and experimental parkinsonism [24]. We therefore evaluated the effectiveness of systemic administration of ODQ to reverse the STN hyperactivity resulting from chronic DA depletion in 6-OHDA-lesioned rats (Fig 3). Changes in the metabolic activity of the STN were assessed in sham-operated and 6-OHDA-lesioned rats by means of histochemical staining of cytochrome oxidase (CO-I) activity. All measures were taken by an investigator blind to the experimental condition. As expected, vehicle-treated 6-OHDA-lesioned rats exhibited a significant increase in STN CO-I staining when compared to the vehicle-treated sham-operated control group (Fig 3a–b). Following one systemic administration of ODQ (20 mg/kg, i.p.), the increase in STN metabolic activity was no longer detected in 6-OHDA-lesioned rats (Fig 3a). To further determine whether this reversal effect of ODQ was mediated via inhibition of striatopallidal output (see Fig 2f), another cohort of sham and 6-OHDA-lesioned rats was generated to examine the effect of intrastriatal ODQ administration on STN metabolic activity. Histological examination of cannula tracks revealed that all intrastriatal microinjections were within the dorsal striatum, between 0.7 to −0.3 mm from bregma (see Methods for details). We found that intrastriatal infusion of ODQ (50 µM) was sufficient to normalize the increase of STN CO-I staining observed in the 6-OHDA group (Fig 3b). Collectively, these observations point to the involvement of the indirect striatopallidal MSNs, as opposed to the direct striatonigral MSNs, in mediating the ODQ-dependent reversal of STN hyperactivity.

**Figure 4:**
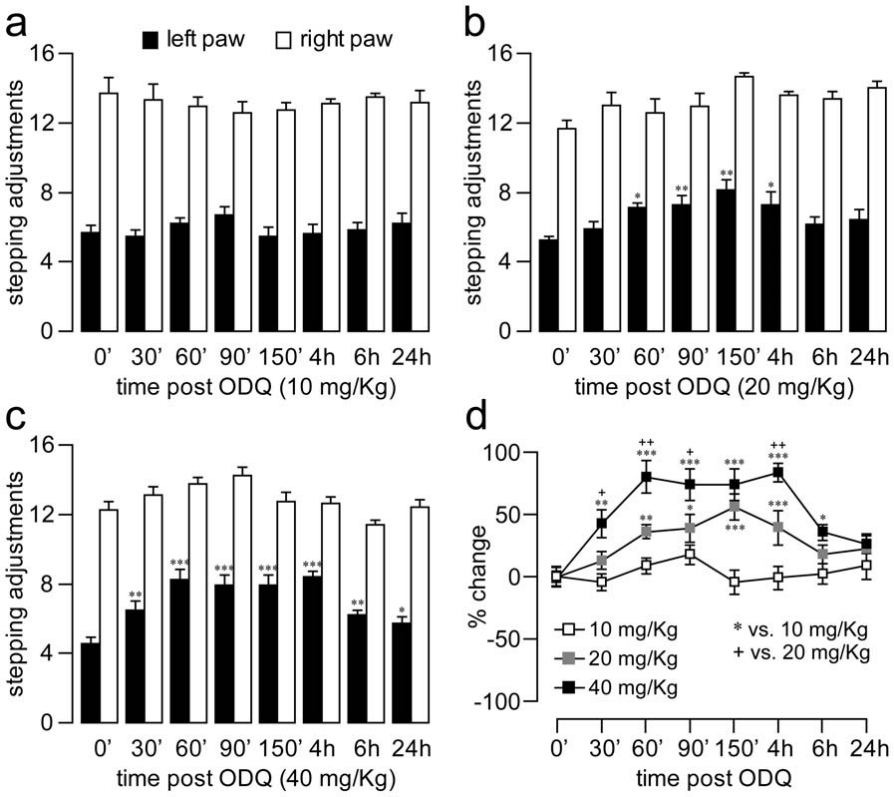
Dose dependent effects of ODQ on reducing forelimb stepping deficits observed in 6-OHDA-lesioned rats. (**a–c**) Bar graphs showing the effect of systemic ODQ administration (10, 20, 40 mg/kg, i.p.) on deficits in forelimb use in 6-OHDA lesioned rats assessed with the stepping test. ODQ treatment transiently attenuated stepping deficits observed in 6-OHDA lesioned rats in a dose-dependent manner (n = 8 rats/group; *P<0.05, **P<0.005, ***P<0.0005, compared to baseline or time 0, Tukey post-hoc test after significant two-way ANOVA). (**d**) Summary graph (% change to baseline or time 0) depicting the impact of ODQ administration (10, 20, 40 mg/kg, i.p.) on deficits in forelimb use observed in 6-OHDA lesioned rats (*,^+^ *P<0.05*, **,^++^ *P<0.005*, ***P<0.0005, compared to measures taken at identical time points as indicated, Tukey post-hoc test after significant two-way ANOVA).

**Figure 5:**
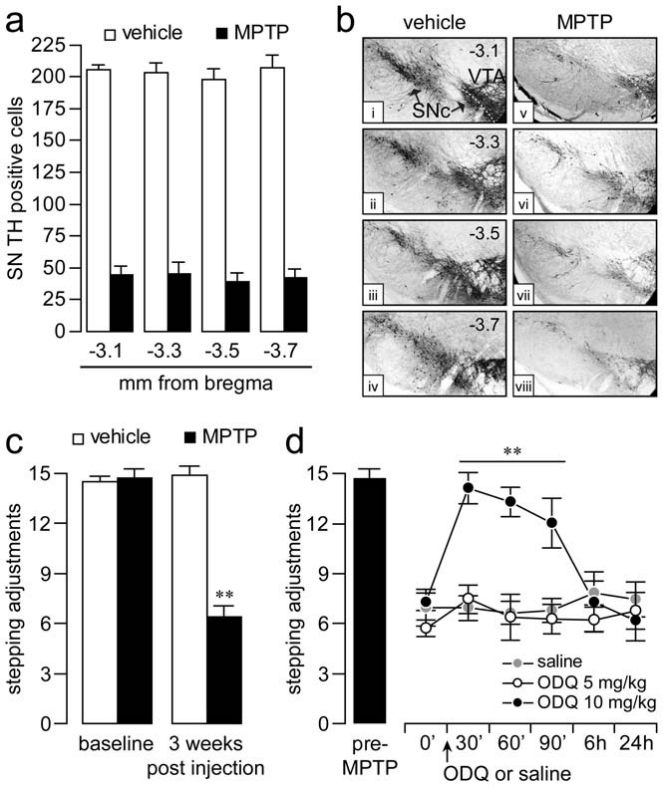
Systemic ODQ administration improves stepping performance in chronic MPTP-treated mice. (**a**) Chronic MPTP treatment significantly reduced the number of TH positive neurons in the SN. The data were collected at four anatomical levels from vehicle or MPTP-treated mice 3 weeks following the last injection (**P<0.005 compared to vehicle). (**b**) Images of TH immunostaining showing the degree of reduction of TH positive cells in the SN at four anatomical levels (mm from bregma): −3.1 (*i, v*), −3.3 (*ii, vi*), −3.5 (*iii, vii*) and −3.7 (*iv, viii*). (**c**) Summary graph depicting the effect of chronic saline (n = 9) or MPTP (20 mg/kg, s.c., n = 13) injections on forelimb stepping performance. A significant decrease in the number of adjusting steps was observed after 6 weeks of MPTP injection (**P<0.005 vs. vehicle, Tukey post-hoc test after significant ANOVA). (**d**) Time course showing the effect of a single s.c. injection of vehicle and ODQ (5 and 10 mg/kg) on MPTP-induced stepping deficits (n = 5–7 mice per group). A transient improvement in stepping performance was detected 30–90 min after ODQ administration (**P<0.005, compared to measures taken at time 0 or identical time points in vehicle, Tukey post-hoc test after significant two-way ANOVA).

We next determined the behavioral significance of ODQ administration by measuring changes in forelimb akinesia in 6-OHDA-lesioned rats. Forelimb akinesia can be quantified in animal models of PD by means of the stepping test, which assesses behavioral parameters thought to resemble limb akinesia and gait problems seen in PD patients [25], [26], [27]. All 6-OHDA-lesioned rats included in this study exhibited similar stepping deficits (Fig 4a–c). We found that the same acute ODQ treatment used in the above biochemical, electrophysiological, and histochemical studies was also effective in reducing forelimb stepping deficits observed in 6-OHDA-lesioned rats. Moreover, the anti-akinetic effects of ODQ were found to be dose-dependent in nature, as revealed by the magnitude and duration of stepping improvement observed with increasing doses of ODQ (Fig 4d). Next, we assessed the effect of ODQ administration on stepping deficits induced by DA depletion in the chronic MPTP mouse model of PD. The MPTP model was chosen because systemic administration of this toxin induces a bilateral DA lesion that reproduces the human parkinsonian state more accurately than that obtained with unilateral lesions [28], [29]. Consistent with our previous study [25], when MPTP was administered to 10-month old mice, the average number of adjusting steps observed in the lesioned mice gradually declined throughout the six weeks of intoxication until it reached a steady state that endured >3 weeks after the last MPTP injection. At this time point, the degree of DA cell loss in the SN was ∼80% (Fig 5a–b) and the stepping performance was markedly reduced by ∼60% (Fig 5c). A one-time injection of ODQ (10 mg/kg, s.c.) was sufficient to reverse the reduction in forelimb stepping behavior observed in MPTP-lesioned mice (Fig 5d) in a manner that was similar to that observed with acute injection of L-DOPA [25]. No apparent improvement in the number of stepping adjustments was observed with saline injection or with a lower dose of ODQ (5 mg/kg) (Fig 5d). Together, these findings attest to the effectiveness of systemic administration of the selective sGC inhibitor ODQ in reversing DA depletion-induced akinesia in two well characterized experimental models of parkinsonism.

## Discussion

We have uncovered an important role of the sGC-cGMP signaling pathway in the regulation of normal corticostriatal transmission and basal ganglia dysfunction induced by chronic DA depletion. We found that systemic administration of the selective sGC inhibitor ODQ decreased corticostriatal transmission in naïve rats in a manner that was reversed by intrastriatal infusion of a cGMP analogue. Moreover, ODQ administration markedly reversed the abnormal elevation in cGMP levels and reduced the increase in spontaneous firing observed in the DA-depleted striatum. These effects of ODQ were also associated with a normalization of the characteristic increase in metabolic activity observed in the STN following nigrostriatal DA cell loss. Moreover, the effects of systemically delivered ODQ on STN hyperactivity were replicated in studies using intrastriatal microinjections of this drug, indicating that striatal sGC inhibition leads to downregulation of striatopallidal output. The effects of ODQ on neuronal hyperactivity in the striatum and the STN were found to be behaviorally relevant as a similar systemic treatment transiently attenuated the reduction in forelimb use observed in 6-OHDA-lesioned rats and mice chronically treated with MPTP. These observations, along with previous studies [14], [15], provide strong evidence that an upregulation of sGC-cGMP signaling in the DA-depleted striatum may contribute to the enduring changes in neuronal excitability and locomotor activity observed in parkinsonian animals. Furthermore, our data demonstrate for the first time that pharmacological attenuation of striatal sGC-cGMP signaling represents a promising novel non-dopaminergic therapeutic approach for restoring basal ganglia dysfunction and subduing motor symptoms associated with PD.

Presently, little is known as to how attenuation of striatal sGC-cGMP signaling may rescue dysfunctional basal ganglia output and behavioral abnormalities associated with experimental parkinsonism. However, converging evidence now indicates that striatal sGC-cGMP signaling plays a key role in the regulation of MSN excitability [12], [17], short and long-term corticostriatal synaptic plasticity [11], [12], [30], [31], [32], and neuronal synchrony [12], [33], [34], [35]. The current studies examining the effects of ODQ on cortically-evoked striatal synaptic potentials in naïve rats indicate that tonic cGMP signaling also facilitates corticostriatal transmission within striatal networks. Taken together with previous studies [12], [16], [17], these findings show that transient elevations in intracellular cGMP markedly increase striatal MSN excitability and facilitate corticostriatal excitatory synaptic transmission. Notably, acute D2 (but not D1) receptor blockade mimics the facilitatory effect of DA depletion on striatal sGC activity [36], [37] and MSN activity [38]. Thus, despite that striatal MSNs from both the direct and indirect output pathways express high levels of all components of the sGC-cGMP second messenger cascade [6], [7], the above studies suggest that alterations in cGMP signaling observed after striatal DA-depletion could result from a preferential upregulation of cGMP synthesis in D2 receptor-expressing striatopallidal neurons of the indirect pathway [16].

Importantly, intrastriatal infusion of ODQ was sufficient to normalize the increased metabolic activity observed in the STN of 6-OHDA-lesioned rats. This finding is of great translational value as STN hyperactivity is one of the pathophysiological hallmarks of parkinsonism that has been repeatedly reported in animal models and in PD. In addition to the indirect pathway (i.e., striatopallidal neurons), there are other afferents known to contribute to the STN hyperactivity such as the excitatory inputs from the parafascicular nucleus of the thalamus and the pedunculopontine nucleus in the brainstem. However, the above outcomes from studies employing intrastriatal ODQ infusions point to a primary role of the indirect pathway in mediating both the STN hyperactivity and the ODQ-dependent reversal of this pathophysiological state induced following DA depletion.

At the cellular level, even less is known regarding how alterations in striatal sGC-cGMP signaling contribute to dysregulation of corticostriatal-striatopallidal transmission observed in the DA-depleted striatum. Given the current findings and previous reports [12], [17], it is possible that inhibition of the sGC-cGMP signaling pathway reduces the abnormal increase in intrinsic excitability observed in striatal MSNs following chronic DA depletion. Indeed, under DA-depleted conditions pharmacological downregulation of sGC-cGMP signaling (i.e., following ODQ administration) may preferentially affect striatopallidal neurons because they are likely to exhibit increased cyclic nucleotide production and PKA/PKG/DARPP-32 activation as a result of decreased D2 receptor-mediated suppression of adenylate cyclase and sGC activity [10]. This prediction is consistent with previous studies showing that drugs that augment cAMP (i.e., the adenylate cyclase activator forskolin) or cGMP (i.e., the phosphodiesterase inhibitor zaprinast) levels in MSNs increase the excitatory impact of corticostriatal transmission on these cells [17], [39]. Activation of the sGC-cGMP signaling pathway is also known to stimulate presynaptic facilitation of glutamate release [40], and to increase surface expression of AMPA receptors at postsynaptic sites [41]. Taken together, these observations indicate that concurrent downregulation of pre- and postsynaptic sGC-cGMP signaling at corticostriatal synapses may be sufficient to normalize the abnormally augmented corticostriatal-striatopallidal transmission observed following DA depletion.

Unveiling the role of non-dopaminergic neural systems in the pathophysiology of experimental parkinsonism has great translational value, as this will open new avenues for treating PD and other debilitating neurological disorders. For instance, given the results of the current study, it is very likely that drugs designed to stimulate metabolism of excessive cGMP (and possibly cAMP) via activation of one or more of the numerous isoforms of phosphodiesterases expressed in the striatum [42], [43] will maximize the specificity of this novel treatment approach. In support of this, in the current study we demonstrated that a second messenger-based therapy (i.e., sGC inhibition and decreased cGMP signaling) is effective for reversing basal ganglia dysfunction and akinesia induced following DA depletion. These observations should lead to a broader understanding of how cyclic nucleotide signaling cascades can be modulated as an approach for treating motor symptoms associated with PD and related neurological disorders. Future studies will have to determine whether an enduring reversal of parkinsonian symptoms can be achieved with a treatment regimen designed to chronically downregulate striatal sGC-cGMP signaling. Moreover, novel studies examining the potential utility of combination therapy using low doses of L-DOPA and inhibitors of sGC-cGMP-PKG signaling are also warranted.

## Materials and Methods

All experimental procedures met the NIH guidelines for the care and use of laboratory animals and were approved by the Rosalind Franklin University of Medicine and Science Institutional Animal Care and Use Committee (Animal Welfare Assurance Number A3279-01, protocols 08-01 and 10–19). All animals were kept under conditions of constant temperature (21–23°C) and maintained on a 12∶12 hour light/dark cycle with food and water available *ad libitum*. All chemicals were purchased from Sigma-Aldrich (St. Louis, MO) whereas ODQ was purchased from Tocris Bioscience (Ellisville, MO).

### Drug preparation

In studies involving systemic drug administration, ODQ (10–40 mg/kg) was dissolved in vehicle consisting of 10% Cremophor EL in 0.9% saline [12]. In studies using striatal ODQ microinjections, ODQ was dissolved in 0.5% DMSO in artificial cerebral spinal fluid (aCSF) and infused into the striatum (see below) at a concentration of 0.05 nmol/µL. The cGMP analog 8-Br-cGMP was dissolved in aCSF [17] and infused into the striatum (0.1 µl/min for 10 min) at a concentration of 20nmol/µL as previously described [43]–[46].

### Local field potential recordings of cortical-evoked striatal postsynaptic potentials in vivo

To determine the specificity of the pharmacological effects of the sGC inhibitor ODQ for downregulation of cGMP and the impact of this attenuation on corticostriatal transmission, we examined the effects of local striatal microinfusions of vehicle or the cGMP analog 8-Br-cGMP on LFPs evoked in the striatum during cortical stimulation. To this end, a concentric bipolar electrode (SNE-100; Better Hospital Equipment, Rockville Centre, NY) was used to stimulate the frontal cortex (B: 2.7 to 3.2 mm, L: 1.5 to 2.2 mm, V: 1.5 mm), while a second concentric bipolar electrode attached to a 28-gauge stainless steel cannula (Plastics One Inc., Reannex, VI) was placed in the dorsal striatum (B: +0.5, L: 3 mm, V: −4.5 mm) to enable the concurrent recording of evoked postsynaptic potentials (PSP) and local microinfusion of vehicle/cGMP analog (Fig 1a). Only naïve adult male Sprague-Dawley (Harlan, Indianapolis, IN) rats (250–350 g) were included in this set of recordings. As previously described [11], [12], [16], animals were deeply anesthetized with urethane (1.5 g/kg, i.p.), placed in a stereotaxic apparatus (ASI instruments, MI) and maintained at 37°C with a heating pad (Vl-20F, Fintronics Inc, Orange, CT). Cortically-evoked striatal PSPs were amplified (NeuroData, Delaware Water Gap, PA), filtered (bandwidth 0.1–100 Hz), digitized (Digidata 1442, Molecular Devices), acquired (Axoscope, Molecular Devices), and analyzed (Clampfit 10, Molecular Devices) at a sampling rate of 10 kHz. The intensity of stimulation (0.5 to 0.9 mA range of single square pulses of 0.3 ms duration delivered every 10 s) was chosen from the minimum amount of current to elicit a PSP with <15% variability in amplitude and slope. The isolated response was therefore monitored for at least 5 min to ensure the stability of the PSP, followed by 15 min of baseline PSP recording. Intrastriatal infusion of the cGMP analog 8-Br-cGMP or vehicle (aCSF) was delivered at the rate of 0.1 µl/min for 10 min using a syringe minipump (BASi Baby Bee Syringe Drives, CA).

### 6-OHDA lesion and stepping test

Adult male Sprague-Dawley (Harlan, Indianapolis, IN) rats weighing ∼250 g were randomly assigned to groups receiving either 6-OHDA or vehicle (sham group). All rats were administered desipramine (10 mg/kg, i.p.) 30 min prior to surgery. Rats were then anesthetized with sodium pentobarbital (55 mg/kg, i.p.) and placed into a stereotaxic apparatus. Eight micrograms of 6-OHDA-free base in 4 µL of 0.1% ascorbic acid was injected unilaterally into the medial forebrain bundle (B: −4.3 mm, L: +1.6 mm, V: −8.3 mm from cortical surface) [18], [27]. The sham lesion was performed by injecting 4 µl of 0.1% ascorbic acid. The injection rate was 0.4 µl/min and the cannula was left in place for an additional 5 min before slowly being removed. Four weeks after surgery, the effect of 6-OHDA on forelimb akinesia was evaluated, as previously described [26], [27]. All biochemical, electrophysiological, histochemical, and behavioral studies were performed >4 weeks after the 6-OHDA or vehicle infusion (average: 46±5 days).

### Quantification of striatal cGMP levels

Rats were decapitated and their brains rapidly excised on an ice-cold surface. Sections containing the striatum were collected and resuspended separately in a chilled solution of 0.1 N HCl, followed by sonication for 15 s. Samples were subsequently centrifuged at 14,000 rpm for 15 min, and the resulting supernatant was collected and stored at −80°C until further processing. The Direct cGMP ELISA Kit-Non-acetylated Version (NewEast; Malvern, PA) was used to determine the levels of cGMP according to manufacturer's instructions. For each sample, the concentration of cGMP was normalized to its protein content determined with Bio-Rad’s DC Protein Assay (Bio-Rad; Hercules, CA). For each animal, a single value was obtained per striatal sample.

### Single unit recordings of striatal activity in vivo

All *in vivo* electrophysiological recordings were conducted following the same experimental procedure as previously described [11], [12], [16]. Briefly, animals were deeply anesthetized with urethane (1.5 g/kg, i.p.), placed in a stereotaxic apparatus (Narishige International USA Inc) and maintained at 37°C with a heating pad (Vl-20F, Fintronics Inc, Orange, CT). Concurrent recordings of striatal single-unit activity (B: −0.5 to 2.0 mm, L: 2.0 to 3.5 mm [44]) and cortical local field potentials (B: 3.0 to 4.0 mm, L: 1.5 to 2.2 mm lateral [44]) were obtained in all experiments. The signals were amplified (NeuroData, Delaware Water Gap, PA or Multiclamp 700B, Molecular Devices, Sunnyvale, CA), filtered (bandwidth 300–3000 Hz), digitized (Digidata 1322A, Molecular Devices) and acquired (Axoscope, Molecular Devices) at a sampling rate of 20 kHz. The isolated single-unit was typically monitored for at least 5 min to ensure the stability of the firing rate, firing pattern, and spike waveform, followed by 10 min of baseline activity recording. All time series analyses were performed with Statistica 6 (Statsoft Inc., Tulsa, OK); the interspike interval was obtained by means of spike amplitude discrimination (Clampfit 10, Molecular Devices).

### Striatal Microinjections

All surgical procedures performed before striatal microinjections were conducted as described above (see *In vivo* electrophysiology). A single stainless steel cannula (28-gauge, Plastics One Inc., Reannex, VI) was first stereotaxically implanted in the dorsal striatum (B: 0.7 mm, L: 3.0 mm, V: −4.5 mm). At least 30 min after implantation, each rat received an intrastriatal infusion (0.1 µl/min for 10 min) of either vehicle (0.5% DMSO in aCSF) or ODQ (50 µM) and all cannula were left in place for 5 min after completion of the microinjection. The same cannula was then placed in a more caudal and lateral position (B: −0.3 mm), and an identical infusion of vehicle or ODQ was performed 20 minutes after completion of the first microinjection. The cannula was left in place for an additional 60 min after completion of the second microinjection, and brains were then extracted for cytochrome oxidase histochemistry.

### Cytochrome oxidase histochemistry and densitometry measures

Histochemistry of CO-I was performed according to a modified protocol of Tseng *et al* (2006) [45]. Coronal sections (50 µm thick) containing the STN were mounted onto glass slides and incubated for 90 minutes at 37°C in 0.1 M PB (pH 7.4) containing 0.50 g/L of 3,3′-diaminobenzidine, 0.33 g/L of horse heart cytochrome c, 44 g/L of sucrose, and 0.2 g/L of catalase. After a 90- min incubation, slides were rinsed, dehydrated and coverslipped. Stained sections were captured with a slide scanner (Coolscan IV; Nikon, Japan), and the mean relative optical density (ROD) per pixel was determined by subtracting the optical density of the background from that of the STN. Background OD was measured at the level of the internal capsule. For each animal, a single value per STN was obtained by averaging measurements from 4–6 sections.

### MPTP model and stepping test

Ten-month old C57BL/6 male mice weighing 25–35 g were single housed with food pellets and water available *ad libitum*. The animal room was maintained at a constant temperature and humidity on a 12 hour light-dark cycle. All animals were acclimated to the animal facility for at least 3 weeks before their use. A baseline stepping performance was monitored in all animals for 2 to 3 days before the first injection, and at least 3 trials of the stepping test were obtained before they were randomly assigned to receive weekly injections of MPTP or vehicle. Each mouse received 2 injections per week (3.5 day intervals) of MPTP (20 mg/kg, s.c.) or vehicle for 6 weeks (12 injections), and subsequent changes in the number of adjusting steps were recorded. The stepping test was performed as previously described [25].

### Tyrosine hydroxylase immunohistochemistry and dopamine cell counting

The extent of the DA lesion was estimated by means of TH immunohistochemistry performed on free-floating sections, as previously reported [25], [27]. Briefly, serial coronal sections of 50 µm thick were obtained from the mesencephalon using a freezing microtome (SM-2000R; Leica Microsystems, Germany) and exposed to rabbit anti-TH for 72 hours. Sections were then incubated for 2 hours in biotinylated goat anti-rabbit IgG (1∶500; Vector Laboratories, CA), and the bound antigen-antibody complexes were visualized with 3,3′-diaminobenzidine and H_2_O_2_ tablets dissolved in 0.05 M Tris, pH 7. The degree of the DA lesion was estimated by counting the number of TH positive neurons in the SN at four serial coronal sections (200 µm apart) using ImageJ (NIH, USA, http://rsb.info.nih.gov/ij/). The 4 stereotaxic planes lie between −5.2 and −5.8 mm from bregma in rats and −3.1 to −3.7 mm from bregma in mice.

### Statistical analyses

All measurements are expressed as mean ± SEM and the differences between experimental conditions were considered statistically significant when P<0.05. In cases where data were not normally distributed or had unequal variances, Kruskal-Wallis ANOVA by ranks was used for multiple comparisons involving interrelated proportions.
